# Identifying Links Between Productivity and Biobehavioral Rhythms Modeled From Multimodal Sensor Streams: Exploratory Quantitative Study

**DOI:** 10.2196/47194

**Published:** 2024-04-18

**Authors:** Runze Yan, Xinwen Liu, Janine M Dutcher, Michael J Tumminia, Daniella Villalba, Sheldon Cohen, John D Creswell, Kasey Creswell, Jennifer Mankoff, Anind K Dey, Afsaneh Doryab

**Affiliations:** 1 University of Virginia Charlottesville, VA United States; 2 Carnegie Mellon University Pittsburgh, PA United States; 3 University of Pittsburgh Pittsburgh, PA United States; 4 University of Washington Seattle, WA United States

**Keywords:** biobehavioral rhythms, productivity, computational modeling, mobile sensing, mobile phone

## Abstract

**Background:**

Biobehavioral rhythms are biological, behavioral, and psychosocial processes with repeating cycles. Abnormal rhythms have been linked to various health issues, such as sleep disorders, obesity, and depression.

**Objective:**

This study aims to identify links between productivity and biobehavioral rhythms modeled from passively collected mobile data streams.

**Methods:**

In this study, we used a multimodal mobile sensing data set consisting of data collected from smartphones and Fitbits worn by 188 college students over a continuous period of 16 weeks. The participants reported their self-evaluated daily productivity score (ranging from 0 to 4) during weeks 1, 6, and 15. To analyze the data, we modeled cyclic human behavior patterns based on multimodal mobile sensing data gathered during weeks 1, 6, 15, and the adjacent weeks. Our methodology resulted in the creation of a rhythm model for each sensor feature. Additionally, we developed a correlation-based approach to identify connections between rhythm stability and high or low productivity levels.

**Results:**

Differences exist in the biobehavioral rhythms of high- and low-productivity students, with those demonstrating greater rhythm stability also exhibiting higher productivity levels. Notably, a negative correlation (C=–0.16) was observed between productivity and the SE of the phase for the 24-hour period during week 1, with a higher SE indicative of lower rhythm stability.

**Conclusions:**

Modeling biobehavioral rhythms has the potential to quantify and forecast productivity. The findings have implications for building novel cyber-human systems that align with human beings’ biobehavioral rhythms to improve health, well-being, and work performance.

## Introduction

### Background

Biobehavioral rhythms—repeated cycles of biological, behavioral, and psychological events—are indicative of different life and health outcomes [1]. Chronobiology, which examines periodic phenomena in living organisms, has demonstrated the impact of circadian disruptions on people’s lives, including physical and mental health as well as safety and work performance in shift workers [2-6]. However, research in chronobiology has primarily been conducted via manual observations and subjective reports often restricted over a small period of time. Advances in mobile and wearable devices provide the possibility of automatic and rigorous collection of longitudinal biobehavioral data from people’s personal devices [7-9]. This longitudinal fine-grained data collected on a daily basis have the potential to reveal micro- and macrolevel patterns related to different biobehavioral outcomes.

In this study, we examine the relationship between cyclical human behaviors and work efficiency using data from mobile sensors. This analysis is based on data collected from the smartphones and Fitbits of 166 college students, encompassing patterns such as activity, communication, and sleep. Our main objective is to determine variations in biobehavioral rhythms across students of varying productivity levels and identify particular rhythm traits associated with productivity.

### Related Work

#### Modeling Biobehavioral Rhythms

Research in chronobiology that examines periodic phenomena in living organisms is relatively mature, and existing studies have confirmed that exploring human rhythms is an effective way to diagnose and treat many illnesses such as cancer, cardiovascular disease, and mental health problems [10-12]. For example, patients with depression, those with bipolar disorder, and those with schizophrenia usually exhibit irregular changes in circadian rhythm, and adjusting the circadian rhythm is an efficient auxiliary method for treating these conditions [13-15]. Disruption in biological rhythms is also caused by changing lifestyles and environmental conditions such as travel across time zones and shift work [16]. Night shift and morning shift workers may be especially at risk of committing errors and having accidents [17].

A few studies have used smartphone technology to track circadian patterns. For example, Abdullah et al [18] used patterns of phone usage to identify chronotypes of students (early birds or night owls). Murnane et al [19] aggregated mobile app usage features by body clock time and analyzed the correlation between circadian rhythms in app usage and alertness level. Doryab et al [1] demonstrated modeling of rhythms using data from Fitbit devices in patients with cancer and showed that disruption in circadian rhythms predicts readmission in patients with cancer undergoing treatment. Yan et al [7] further developed a computational framework for modeling biobehavioral rhythms from multimodal sensor streams. While our work leverages this framework to model biobehavioral rhythms, we advance research in this domain by developing and applying algorithms to observe and measure changes in multimodal biobehavioral rhythms across different periods and between people with different productivity levels.

#### Productivity Assessment

Traditional productivity assessment approaches are typically subjective, static evaluations administered as self-report surveys, manager assessments, observations, or ability tests. Some studies have used multitasking and interruptions, for example, checking emails [20] and mental and physical fatigue as proxies for productivity in workers and officers [21-24]. For example, Gloria et al [20] tracked and analyzed email usage in affecting workplace productivity and stress. Aryal et al [25] conducted a simulated construction task for monitoring physical fatigue by measuring changes in heart rate, skin temperature, and brain signals. The study showed a direct relationship between physical fatigue and heart rate metrics such as heart rate, heart rate variability, and percentage of heart rate.

Recent studies on workplace productivity have used mobile, wearable, and environmental sensors to track individuals’ behavior and environmental conditions to assess workers’ job performance. For example, background noise, light, temperature, and air quality have been shown as the 4 external factors affecting productivity [26-29]. In a study by Mirjafari et al [30], the analysis of phone usage, location, activity, sleep, and time allocation of 554 participants indicated that the regularity of behaviors distinguishes high and low performance. van Vugt et al [31] suggested that eye-tracking could be used to measure productivity. The hypothesis was that if the eyes of a person remained at certain locations on the computer screen, they were focused and thus productive. However, this theory has yet to be evaluated in practice. In addition to external factors, research studies have investigated the impact of internal factors and cues in measuring productivity. For example, Das Swain et al [32] demonstrated that static intrinsic personality can explain workplace performance using data from 603 information workers.

Our research is unique in measuring and assessing productivity by leveraging cyclic biobehavioral patterns from passive data streams to assess productivity. Our work is also the first to measure daily productivity from multimodal mobile and wearable data in college students.

## Methods

### Data Collection

We use a data set of smartphone and Fitbit logs collected from 188 students at an American university over the course of 1 semester. The data were collected as part of an extensive study on students’ health and well-being. All participants were first-year students, with their demographic details presented in Table 1.

The AWARE data collection app [33] and Fitbit were used for the collection of audio, Bluetooth, Wi-Fi, location, phone usage, calls, calories, sleep, and steps. AWARE is an open-source data collection framework that works both on Android and iOS platforms. All participants used their smartphones, and this study’s team provided a Fitbit Flex 2 to collect data. Students’ productivity assessments were collected via an evening survey during weeks 1, 6 (midsemester), and 15 (last week of classes) of the semesters to avoid overburdening participants. The assessment question included a single question: “How productive did you feel today?” The possible responses ranged from 0 (not productive at all) to 4 (extremely productive). The mean and SD of self-evaluated productivity scores were consistent for different sexes and major groups with no significant difference: female (mean 1.65, SD 0.92), male (mean 1.80, SD 0.97), engineering (mean 1.71, SD 0.96), business (mean 1.70, SD 0.99), science (mean 1.69, SD 0.94), art (mean 1.76, SD 0.95), humanities (mean 1.68, SD 0.97), and undecided (mean 1.67, SD 0.87).

Of the initial 188 first-year students, 166 produced subjective assessments of their respective daily productivity. The response rate fluctuated over the 3 weeks, with some students not completing the surveys. The data set included 488 total observations, represented as participant-week pairs. During the introductory meeting, students were briefed about this study’s objectives. This study’s goals were transparently communicated without any deceit or exclusion.

**Table 1:** Demographic distribution of this study’s samples: a total of 188 first-year university students were enlisted as participants for this research.

Category and subcategory		Participants, n (%)
**Sex**		
	Male	111 (59)
	Female	77 (41)
**Race**		
	Asian	107 (57)
	Black	9 (5)
	Hispanic	17 (9)
	White	64 (34)
**Major**		
	Engineering	79 (42)
	Art	30 (16)
	Business	24 (13)
	Science	23 (12)
	Humanities	8 (4)

### Data Processing

#### Measuring Productivity Levels

**Figure 1:**
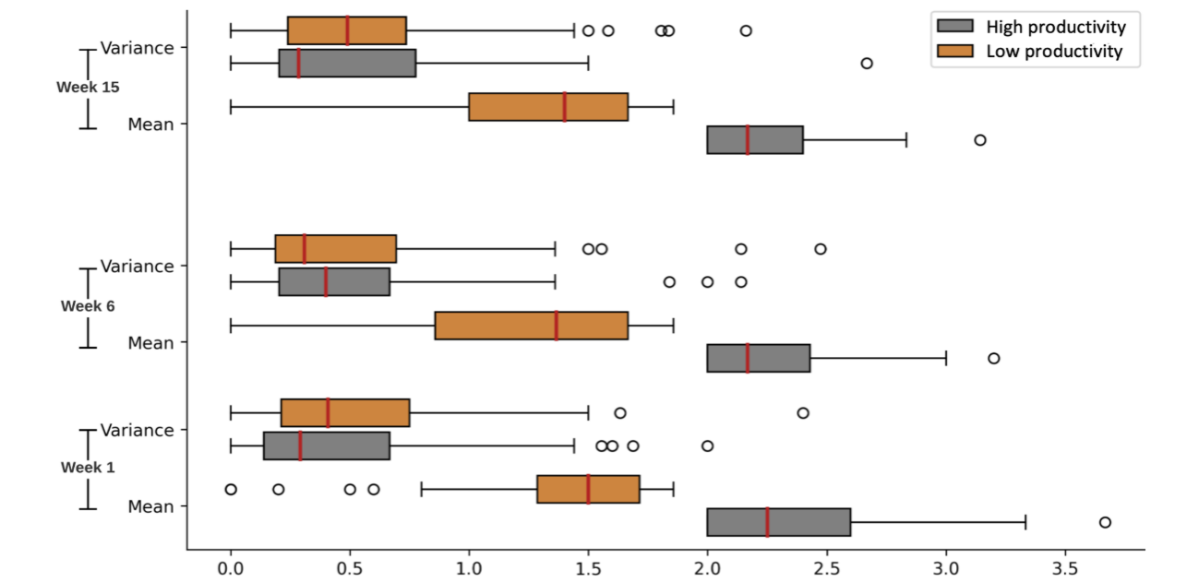
If the mean of 1 week’s daily productivity is above 2 (SD 0.21), the week will be labeled high productivity; otherwise, the week will be labeled low productivity. Gray represents the mean and variance that come from weeks with high productivity, and orange represents the mean and variance that come from weeks with low productivity. The medians of variance are all less than 0.5, and the 75 percentiles are within 1 no matter what productivity the weeks have. The difference in productivity scores between the 2 adjacent options in the productivity survey is 1, so the low variance indicates that most participants will keep the same productivity level during the whole week. The medians of the mean of both high and low productivity are very close, but there are more small mean values in week 6 for low productivity and more large mean values in week 1 for high productivity.

As mentioned previously, while sensor data were collected continuously for 16 weeks, self-reported productivity (by study design) was only collected in weeks 1, 6, and 15. We used productivity scores (0-4) to categorize participants into high and low-productivity groups. These categories were used as ground truth labels in the later analysis of the relationship between rhythms and productivity. To identify the cutoff threshold, we calculated the mean and median of the daily productivity scores for all participants across all 3 weeks. The mean of 1.89 (SD 0.94) and a median of 2 (IQR 1) indicated a normal distribution across scores (verified by the Shapiro-Wilk test, *P*=.12). Therefore, we used 2 as the threshold for categorizing productivity, with scores less than 2 indicating low productivity and scores equal to or above 2 indicating high productivity. Figure 1 shows the distribution of the mean and variance of daily productivity scores within each week. The mean productivity has decreased in week 6 compared with week 1. Since week 6 is the midterm, a high workload and pressure may make some students work more productively, but the pressure and stress may have the opposite effect on others. The IQR of the mean of low productivity is wider than in week 1. The mean and 75th percentile of variance are all less than one, which is also the interval between the survey’s productivity options. This indicates that the participants’ answers are relatively stable each week. We, therefore, average the productivity scores of all days in each week (including both weekdays and weekends) as the weekly productivity score with the same threshold to categorize each participant’s week average into high or low productivity.

In addition to labeling each participant’s weekly data as high or low productivity, we also need to further categorize participants into high or low productivity to evaluate our rhythm similarity methods described in the Methods section. We analyze the combination of high and low productivity weeks for all participants as shown in Table 2. We observe the number of participants in different combinations is imbalanced and does not create large enough groups for analyzing each combination separately. We therefore categorize participants into high and low productivity groups, where students with at least 2 weeks of high productivity rates are categorized as high productivity and the rest are placed into the low-productivity group.

**Table 2:** Participant productivity^a^.

Week 1		Week 6	Week 15	Participants, n
**High productivity group**				
	High	High	High	13
	High	High	Low	14
	High	Low	High	12
	Low	High	High	10
**Low productivity group**				
	High	Low	Low	29
	Low	High	Low	4
	Low	Low	High	17
	Low	Low	Low	62

^a^The middle column lists all combinations of weekly productivity levels, and the right column shows the number of participants for each combination. Many participants were inefficient for all 3 weeks. Participants were more likely to achieve high productivity in week 1 and had the most difficulty achieving high productivity in week 6. Moreover, we aggregated the 8 combinations into 2 groups. Participants with at least 2 highly productive weeks were assigned to the high-productivity group; otherwise, they were assigned to the low-productivity group.

#### Feature Extraction

We extracted features in 2 processing layers. First, we aggregated the raw sensor data into more meaningful behavioral features to capture students’ social interaction, physical activity, sleep, and academic life. The raw sensor data we collect are just a series of numbers without providing much information. For example, screen data are a time series of values from 0 to 3 (eg, 0121023...), which does not provide any helpful information, but we can process this time series to extract more meaningful information about how often the user has been interacting with the phone. We then divided each data stream into hourly intervals and extracted behavioral features in each interval following the descriptions documented by Doryab et al [34]. Typical features included statistical measures such as minimum, maximum, mean, SD, length of the status in the hour, and more complex behavioral features such as movement patterns and type and duration of activities. Example features are shown in Table 3. Finally, we modeled the cyclic pattern of each behavioral feature using Cosinor, which provided a set of parameters that describe the cyclic pattern. This process and the list of rhythm parameters are detailed in the following section.

**Table 3:** Examples of sensor features.

Device and sensor		Extracted feature
**Smartphone**		
	Audio	Percentage of time with voice, noise, or silence; minimum, maximum, mean, or SD of voice energy
	Bluetooth	Mean or total number of Bluetooth scans
	Wi-Fi	Number of unique Wi-Fi hotspots detected
	Location	Location variance; percentage of time staying at home; number of visits; time spent at green areas, athletic areas, academic areas, or outside campus
	Phone usage	Minutes interacting with phone; minimum, maximum, mean, or SD length of interaction periods
**Fitbit**		
	Sleep	Minutes asleep, awake, or restless; minimum, maximum, or mean length of asleep, awake, or restless periods
	Steps	Total number of steps; minimum, maximum, mean, and total length of active or sedentary periods
	Calories	Minimum, maximum, mean, or total calories burned; minimum, maximum, mean, or total decrease in 5-minute calories burned

#### Handling Missing Values

As data sets collected in the wild are expected to include noise and missing data, we developed strategies to handle missing data. The missing values were filled separately for different participants and weeks using the local moving average commonly used in time series. For example, if the hourly values of location variance were missing at 2 PM and 3 PM on day 1 of week 1 for participant A, then we imputed the values as follows: *v_2pm_* = *v_1pm_* + (*v_4pm_* – *v_1pm_*) / (4 – 1) and *v_3pm_* = *v_1pm_* + 2 × (*v_4pm_* – *v_1pm_*) / (4 – 1). Moving average is the most suitable interpolation method for rhythm modeling. Other methods such as multiple interpolations and Expectation-Maximization estimation introduce cross-correlation between features, and regression estimation and k-nearest neighbor increase auto-correlation of a single sensor feature [35,36]. However, the moving average method is sensitive to the number of continuous missing data. If the missing block is large, the moving average will introduce high noise and bias, and the data may need to be removed instead of imputed. We, therefore, calculated the average length of continuous missing hour blocks to decide the minimum threshold for removing data. The average missing block was 1.7 (SD 0.41) data points in sensor streams with less than 20% missing values. We, therefore, imputed the behavioral feature streams with less than 20% missing values and discarded the rest.

After cleaning the data, we ended up with a data set that included 101 sensor features related to location, calories, steps, and sleep. The amount of weekly data we have for each feature changes because some data from participants was removed during our missing handling process. As an example, location features have around 50 observations for week 1 and 15 and 22 observations for week 6; calories and steps features have around 110 observations for weeks 1 and 6 and 80 observations for week 15.

### Modeling Biobehavioral Rhythms

**Figure 2:**
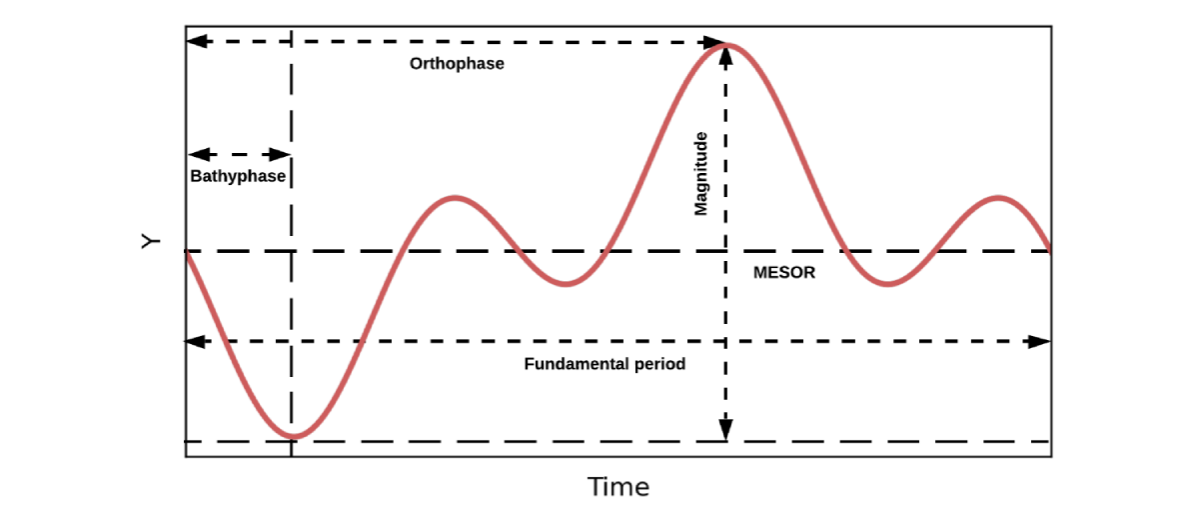
The cyclic wave is formed by fundamental parameters described in Table 3 (adapted from Cornelissen [7]). MESOR: Midline Estimating Statistic of Rhythm.

To model rhythms from longitudinal biobehavioral data collected in the wild, we used the Cosinor method introduced by Halberg [37]. The Cosinor method forms a linear combination of cosine curves with known frequencies to fit cyclic time-series rhythm data and calculates rhythm parameters using least square regression [38]. The Cosinor function can take multiple periods as input parameters and use those to generate a cyclic model of provided time series data. The generated model includes a series of parameters that characterize the cyclic behavior in the data stream. Textbox 1 details the parameters, and Figure 2 [39] visually represents them. The Cosinor method is mathematically expressed by Fernández et al [40] as:



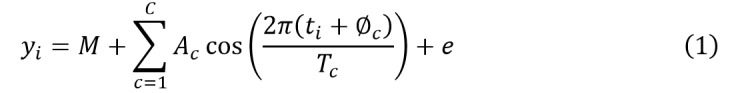



where *y_i_* is the observation at time *t_i_*; 𝑀 is the Midline Estimating Statistic of Rhythm (MESOR); *t_i_* is the sampling time; 𝐶 is the number of input periods; *A_c_*, *T_c_*, and *Ø_c_* represent the amplitude (Amp), period, and acrophase (PHI), respectively; and is the error. Cosinor also outputs the SE for MESOR, Amp, and PHI, respectively.

We used Cosinor to build personal cyclic models per student per sensor stream in weeks 1, 6, 15, and the weeks adjacent to them (eg, for week 6, we use sensor data from weeks 5, 6, and 7 to build Cosinor models). We then used rhythm parameters generated by those models in the correlation analysis. We assumed all participants had normal daily rhythms and used the input periods of 8, 12, and 24 hours in the Cosinor. The 8, 12, and 24 hours reflect nocturnal, diurnal, and circadian duration, respectively.

**Definitions of rhythm parameters output from the Cosinor model [41].**

**Rhythm parameters and their definition**

- Fundamental period: the fundamental period is the least common multiple (LCM) of all individual periods. We use 8-, 12-, and 24-hour periods in our modeling approach.
- MESOR: estimating the midline of the rhythm curves.
- Amplitude (Amp): half the difference between the maximum and the minimum of the best-fitted curve in an individual period.
- Acrophase (PHI): lag from a defined reference time point to the maximum point within an individual period.
- Magnitude: half the difference between the maximum and the minimum of the best-fitted curve in the fundamental period.
- Bathyphase: lag from a defined reference time point to the minimum point within an individual period.
- Orthophase: lag from a defined reference time point to the maximum point within the fundamental period.
- *P* value (P): *P* value indicates the significance level of the model fitted by an individual period.
- Percent rhythm (PR): percent rhythm is the coefficient of determination (*R*^2^) for the model using an individual period.
- Integrated *P* value (IP): the integrated *P* value indicates the significance level (*P* value) of the model fitted by the fundamental period.
- Integrated percent rhythm (IPR): integrated percent rhythm is the (*R*^2^) for the model using the fundamental period.

### Measuring the Relationship Between Rhythms and Productivity

**Figure 3:**
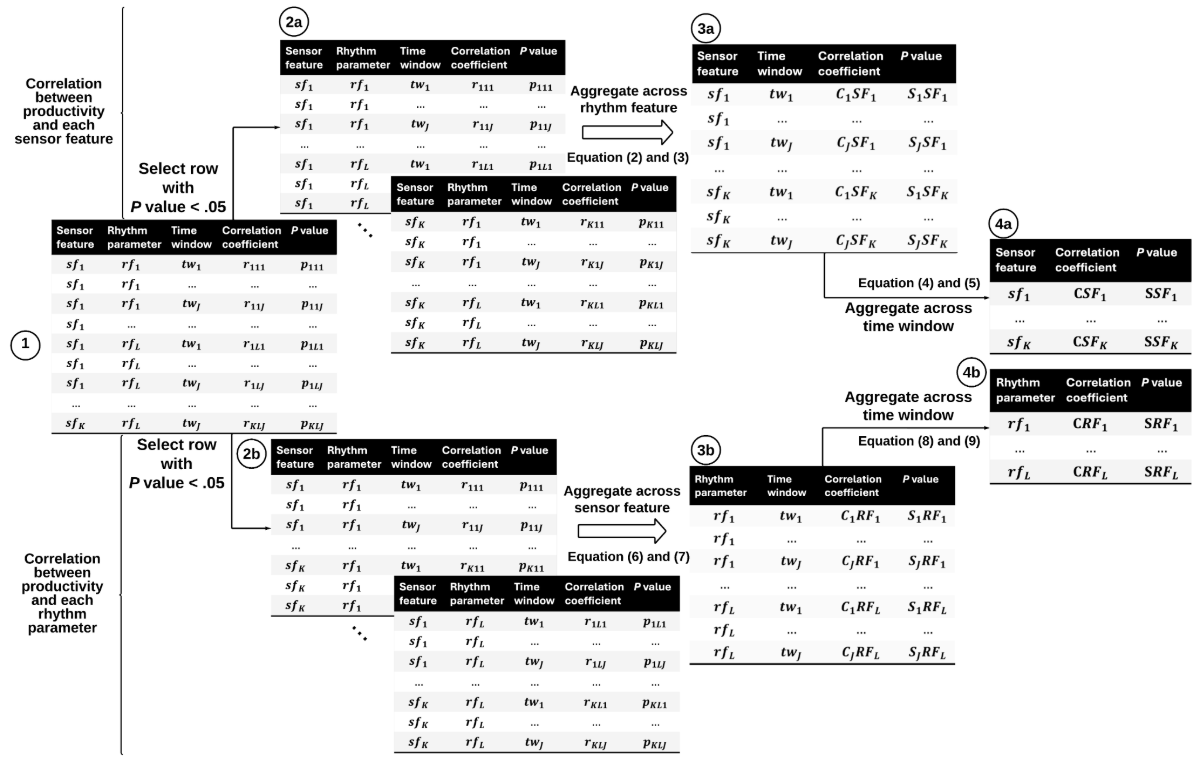
The pipeline to aggregate the correlation for a multidimensional dataset with K sensor features, L rhythm parameters, and J time windows. The pipeline can output the correlation between productivity and a single sensor, and the correlation between productivity and a single rhythm parameter. In step 1, we got a correlation coefficient and a *P* value for each behavior, rhythm setting, and week. In step 2, we calculated how rhythms changed related to productivity for each behavior sensor weekly (2a) and for each rhythm setting weekly (2b). In step 3, we combined the correlation and importance scores from all 3 weeks. Finally, in step 4, we converted the correlation and importance values into 1 final score for each sensor behavior (4a) and each rhythm setting (4b).

We adopted the Pearson correlation analysis to identify relationships between rhythms and productivity across time windows (here weeks). Such a relationship, however, is multidimensional, involving multiple sensors, features, and rhythm parameters. To quantify this multidimensional relationship, we developed a 2-step method. First, we calculated the correlation coefficient between each rhythm parameter and productivity score to understand how rhythm parameters correlate with productivity and whether the correlation is consistent across weeks. To account for the varied scales of productivity and rhythm parameters, we initially applied minimum-maximum normalization to both the productivity scores and each rhythm parameter. Following this, we computed the Pearson correlation coefficient and determined its significance using a 2-tailed *P* value test. The first step resulted in 1 correlation coefficient and 1 *P* value per behavioral feature, per rhythm parameter, and per time window (week) as shown in Figure 3 (step 1). The correlation coefficient indicates how closely the rhythm parameter and productivity score are related, and whether they move together or in opposite ways. The *P* value helps us understand if this relationship is significant or merely coincidental.

Next, as presented in Figure 3, we adopt the Fisher method to combine the correlation coefficient and its significance (*P* value) of every combination of behavioral feature—rhythm parameter—week. The Fisher method is a widely used meta-analysis technique used for combining the results from several independence tests [42,43]. These combinations provide information about productivity-related variations of the rhythms for each behavioral feature per week (2a in Figure 3) and productivity-related variations of each rhythm parameter per week (2b in Figure 3) regardless of behavior. While the correlation coefficient represents the strength and direction of the relationship, its significance reflects the reliability and generalizability of the relationship. We, therefore, aggregated significant correlation coefficients for all rhythm parameters per behavioral sensor feature (2a) as well as aggregated significant correlation coefficients for all sensor features per rhythm parameter per week (2b). In step 3 (3a and 3b), we further combined correlation coefficients and significance scores across all 3 weeks. The final step (4) summarizes the correlation (and significance) values into 1 final score for each sensor feature (4a) and for each rhythm feature (4b). The calculation process is detailed in the Multimedia Appendices 1 and 2. Since the number of observations is different for different rhythm parameters, behavioral sensor features, and weeks due to missing values, this analysis was only performed on the correlations with more than 28 observations, which is the median value in our data set.

### Ethical Considerations

All data collection procedures were approved by an American university’s institutional review board (Carnegie Mellon University; STUDY2016_00000421).

## Results

### Overview

**Figure 4:**
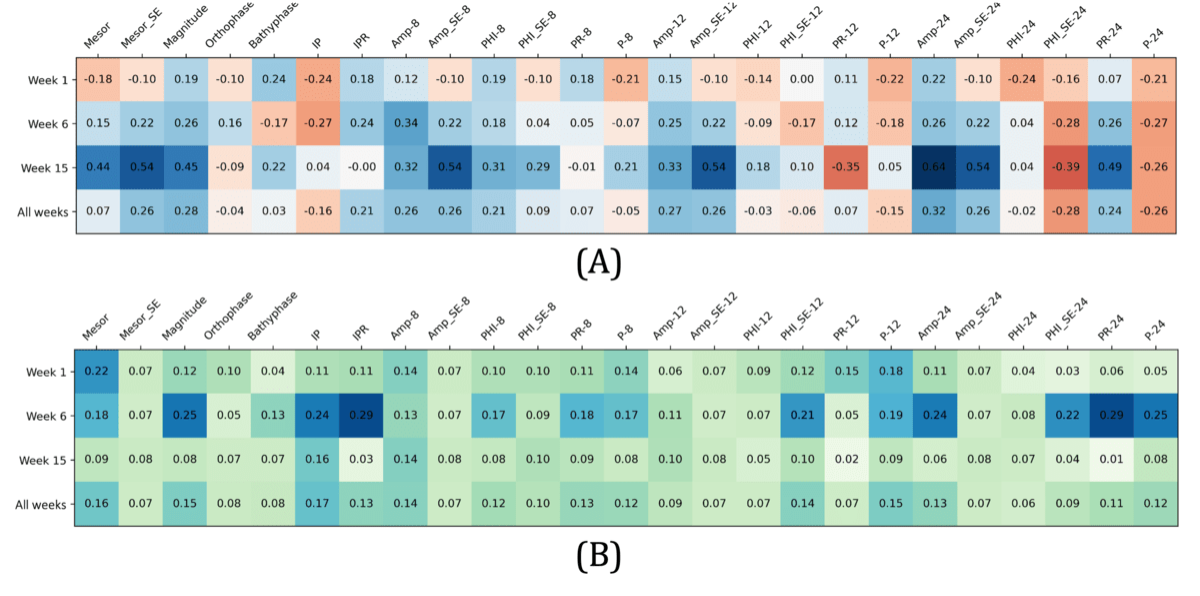
The heat map displays correlations between rhythm parameters and productivity by week. (A) Average correlation coefficients (C-RF) by week (Week-C); (B) Average significance score (S-RF) by week (Week-S). AMP: amplitude; C: correlation coefficients; IP: integrated *P* value; IPR: integrated percent rhythm; MESOR: Midline Statistic of Rhythm; P: *P* value; PHI: acrophase; PR: percent rhythm; RF: random forest; S: significance score.

**Figure 5:**
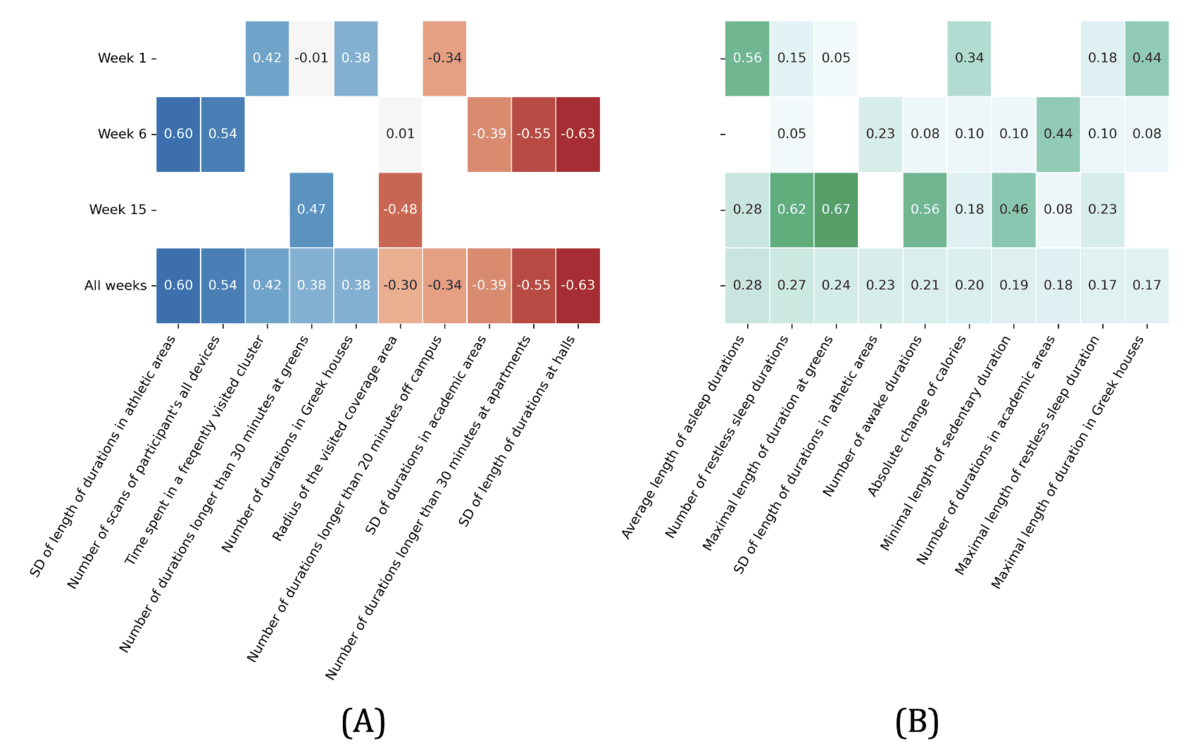
The heat map displays the correlation between sensor features and productivity by week. The left side shows the 10 sensor features with the highest aggregated correlation over all 3 weeks, and the right side shows the 10 sensor features with the highest aggregated significance score over all 3 weeks. The blank cells shown in the figure mean that the relationship is not significant. (A) Average of correlation (C-SF); (B) Significance score of correlation (S-SF). C: correlation coefficients; S: significance score; SF: sensor feature.

While correlations between rhythm parameters and productivity scores were moderate across all behavioral sensor features and all 3 weeks (Figure 4), we observed more pronounced relationships between parameters related to regularity in rhythm models, including SE, that is, deviation of the fitted model parameter from the actual values, percent rhythms (PR and integrated percent rhythm [IPR]) or proportion of variation accounted for by the fitted model, and the significance of the fit (*P* value and integrated *P* value [IP]). In addition, the aggregated negative correlation (indicated by the red line) in the majority of these parameters across all 3 weeks indicates lower rhythm irregularity in highly productive students. The rhythm parameters for location features appeared to be dominant in both aggregated correlation coefficients and significance scores, followed by activity and sleep features (Figure 5). In the following, we discuss our observations in detail.

### Correlation Aggregation of Rhythm Parameters

#### Overview

The blue and red cells in Figure 4 show the correlation aggregated by week for each rhythm parameter as calculated using equations 8, 9, 11, and 13 in Multimedia Appendix 2. Recall that these formulas aggregate correlation across all sensor features for each rhythm parameter to measure the strength of the correlation between productivity and the rhythm parameter. Blue cells indicate a positive correlation while red cells indicate a negative correlation.

The green cells in Figure 4 show the significance score by week for each rhythm parameter as computed by equations 10 and 12 in Multimedia Appendix 2. These formulas calculate correlation significance across all sensor features for each rhythm parameter to measure the significance of the correlation between productivity and the rhythm parameter. The higher the significance score, the more significant the relationship is.

#### Week 1

In week 1, the majority of parameters that measure the irregularity of the rhythm models correlate negatively with productivity indicating more stable rhythms in the high productivity group. For example, stronger correlations were observed between productivity and the model fit for the fundamental period (IP; C=–0.24), the 24-hour period (P-24; C=–0.21), the 12-hour period (P-12; C=–0.22), the 8-hour period (P-8; C=–0.21), the fundamental PR (IPR; C = 0.18), and SE of phase fit for the 24-hour period (PHI_SE-24; C=–0.16).

The relationship between regularity in rhythms and productivity is further reinforced by the negative aggregated correlation coefficients for P-24, P-12, P-8, IP, and SE. Specifically, their low values indicate that Cosinor was able to create close fits to the actual data which means more regularity in the actual data corresponds to high productivity. This further demonstrates a lower rhythm variation in highly productive students.

The relationship between lower rhythm variability and higher productivity is also observed in the correlation of MESOR_SE, Amp_SE-8, Amp_SE-12, Amp_SE-24, and PHI_SE-24. The values have a relatively high aggregated significance score compared to other parameters. This means the SE has a more significant relationship with productivity. Given that the SE is also a metric reflecting the irregularity of rhythm models, its negative correlation indicates less irregularity of the rhythm models in high productivity.

The PR parameter also demonstrated a relationship between low rhythm variability and high productivity. A higher PR represents low variability in the actual data. Specifically, the PR of the fundamental, 24-hour, 12-hour, and 8-hour periods all have high positive aggregated correlation coefficients with productivity, indicating lower variability in diurnal activities for the highly productive students.

#### Week 6

Week 6 (midterm) projected a relatively different pattern. For example, we found positive correlations between productivity and MESOR_SE, Amp_SE-8, Amp_SE-12, and Amp_SE-24. Since Amp and MESOR are indicative of the intensity and volume of activities, we see that highly productive students performed more intense activity during week 6.

We also found Amp and MESOR have higher SE in the fitted models. This implies higher variability in the intensity of regular patterns during this week. This can be expected due to midterm pressure.

Despite this increased variability of intensity of regular activities, as demonstrated by the positive aggregated correlations of IPR (C=0.24) and PR-24 (C=0.26) with productivity, we see less irregularity in activity patterns during this week for the highly productive students.

Finally, as in week 1, we see positive correlations between PR and productivity. However, the correlation became more stable in week 6 compared to week 1 with larger aggregated significance scores.

#### Week 15

Week 15 (the week before finals) showed the strongest correlations. For example, parameters that reflect irregularity in rhythms such as SE (eg, MESOR_SE, Amp_SE, and PHI_SE) show high (mostly positive) correlations with productivity. Parameters characterizing the fitted cyclic model such as MESOR, phase, and Amp also show high (mostly positive) correlations with productivity indicating higher intensity and duration of behavioral activities during this week.

The value of some correlations, however, decreased from weeks 1 and 6 to week 15. For example, the correlation between PRs (eg, IPR, PR_8, and PR_ 12) and productivity. Given the increased workload activities close to final examinations, the observed irregularity and divergence from the routine patterns are expected.

Despite the decline in the value of some correlations, observations across all 3 weeks still suggest an overall lower irregularity in rhythms among the high-productivity group. For example, there is a consistent negative correlation of the regularity indicators such as P-24, P-12, P-8, PHI-SE-24, PHI-SE-12, PHI-SE-8, and IP. Moreover, parameters representing the phase’s characteristics in rhythms including orthophase, bathyphase, PHI-24, PHI-12, and PHI-8 exhibit relatively high aggregated significance scores in all 3 weeks. This means more regularity in phase is more significantly correlated with high productivity. Thus, while further explorations are needed, these observations indicate the importance of rhythm stability in students’ productivity.

### Correlation Aggregation of Sensor Features

#### Overview

Figure 5 shows the aggregated correlation and significance scores by week for the top 10 sensor features calculated through equations 2, 3, 4, 5, 6, and 7 in Multimedia Appendix 1. These formulas calculate the aggregated correlation coefficients and significance scores across all rhythm parameters for each sensor feature to measure the strength of the correlation between productivity and behavioral sensor features. Features with higher significance scores have a more significant correlation with productivity. Overall, location features had a stronger aggregated correlation and significance. The rhythm model for each sensor feature was not consistently associated with productivity in all 3 weeks.

#### Week 1

In week 1, rhythm parameters for both the time spent in frequently visited places and the frequency of visits in fraternity or sorority houses (places for socializing) showed the highest average positive correlations with productivity. A negative correlation between productivity and off-campus duration was also observed in the rhythm models. Finally, we found patterns of asleep and burned calories to have high significance scores.

#### Week 6

In week 6, the variance of the length or number of stays in academic areas, halls, and apartments showed high negative aggregated correlations with productivity (the left side of Figure 5), indicating that highly productive students had a stable living and studying environment at home and school. Conversely, the SD of duration in athletic areas was positively correlated with productivity. This indicates higher variability in exercise associated with high productivity. A similar conclusion can be drawn with the data from the aggregated significance score data (the right side of Figure 5).

#### Week 15

In week 15, we observed the highest aggregated significance scores for rhythms of restless sleep duration, awake sleep duration, time spent at greens, and sedentary duration. On the left side of Figure 5, we see the time spent at greens was positively correlated with productivity, whereas the radius of the visited areas was negatively correlated with productivity. This finding suggests that high-efficiency students reduced their range of activities and spent time outdoors more frequently in week 15.

**Figure 6:**
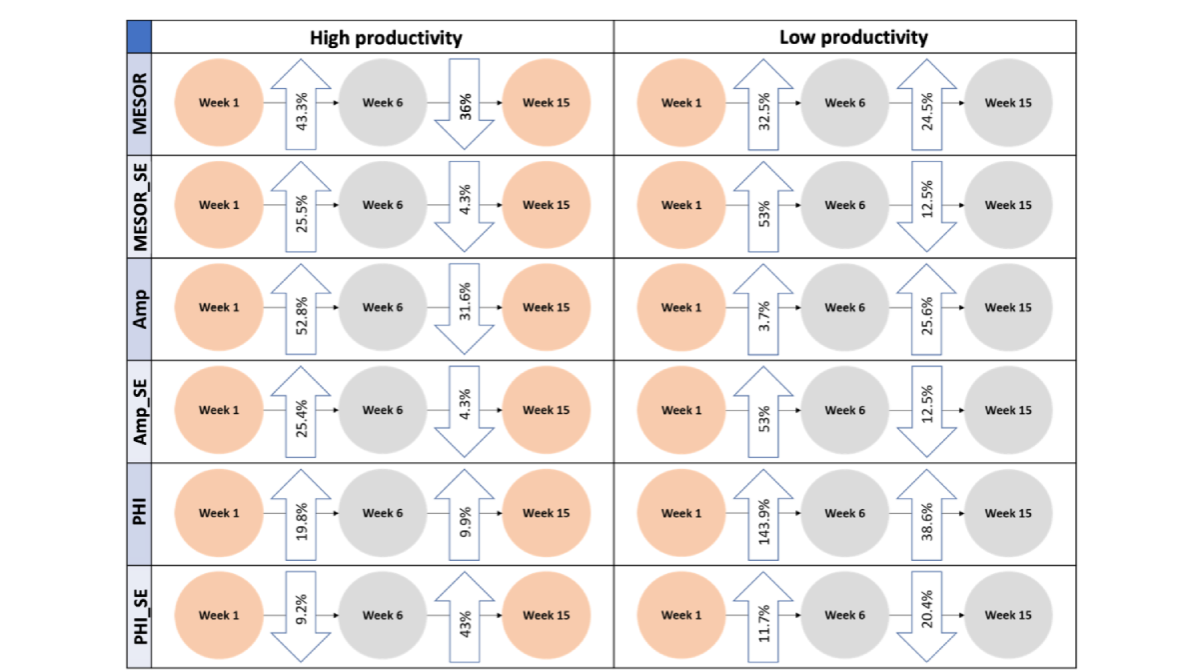
Change in restless sleep and productivity patterns of 2 sample students. Orange and gray represent high and low productivity, respectively. The direction of the arrows indicates an increase or decrease of the rhythm parameter values between weeks. AMP: amplitude; MESOR: Midline Estimating Statistic of Rhythm; PHI: acrophase.

We further select the “restless sleep” feature to visualize how changes in rhythm parameters reflect the change in productivity for 2 individual students in our sample (Figure 6). The left and right columns in the figure show changes in rhythm parameters between weeks for 1 high- and 1 low-productivity student, respectively. While both students’ productivity levels lowered in week 6, their rhythm parameters of MESOR (SE), Amp (SE), and phase increased from week 1 to 6 with substantially higher variations in the parameters of the low-productive student. After week 6, the student’s productivity in the left column went back to high while MESOR and Amp of their restless sleep rhythm substantially lowered. However, the pattern for the student on the right remained relatively unchanged. As the values of these parameters reflect intensity (Amp and MESOR), duration (phase), and variation (SE), the figure shows that an increase in intensity, duration, and irregularity of restless sleep may be indicative of lower productivity in both students. Although we only look at 2 random participants, the positive and negative changes in rhythm parameters and their accordance with changes in productivity pose an interesting observation and call for further exploration.

## Discussion

### Principal Findings

In this paper, we analyze cyclic human behavior using passive multimodal mobile sensing data to understand its correlation with work productivity. By creating rhythmic models for each sensor type and employing a multidimensional correlation-based algorithm, we examine the links between biobehavioral rhythms and daily work performance evaluations. Our data are sourced from smartphones and Fitbits of 166 college students, capturing behaviors such as activity, communication, and sleep patterns. The main aim of our analysis is to identify variations in biobehavioral rhythms based on productivity levels and identify specific rhythmic traits associated with them.

To the best of our knowledge, this study pioneers the modeling relationships between daily productivity and biobehavioral rhythms derived from passive sensor data. Notably, we evaluate the capability to model cyclic behavior from detailed phone and Fitbit data. Additionally, we introduce a novel method to measure the correlation and importance of various sensors and rhythms to productivity, which illuminates the connection between rhythmic consistency and different levels of productivity.

Overall, our results showed more rhythm stability in the high-productivity group of students in our sample despite changes in students’ workload in different weeks. This observation was especially projected by lower variation accounted for in fitted rhythm models (indicated by PRs and SE parameters) and more significant fit levels (indicated by *P* parameters) across the weeks. In addition, our correlation analysis of rhythms for each sensor feature showed the significance of consistent patterns in location and sleep to productivity. While encouraging, these results call for more data and analyses to replicate and improve.

### Limitations

However, this study was not devoid of limitations. A notable constraint was data quality and its lack of completeness. Inherent issues such as device malfunctions, device misplacement, and time zone travels are usual and expected in mobile and sensor data collection studies. These issues were frequently observed in our data set and contributed to different lengths of time series data for each sensor feature in the modeling step. To address this, we employed data imputation and elimination strategies. The longitudinal repeated-measures design of our study helps mitigate the influence of transient noise or anomalies in the data. By modeling everyone’s rhythms across multiple weeks, we reduced the influence of random confounding events. However, we acknowledge that the persistent confounds affecting multiple weeks of data for a given participant could bias their overall rhythms models. We plan to further evaluate our methods on other similar data sets of human behavior such as Tesserae [44], TILES [45], and RAAMPS [46]. We also plan to extend our study to other groups such as construction workers and office staff in the future.

Few other limitations were imposed by the data set we used in this paper, notably its inclusion of only 3 weeks of noncontinuous self-reported productivity covering the beginning, middle, and end of a semester despite continuous sensor data. Although this was deliberately designed to reduce the burden of frequent self-reports, it limited our ability to model the relationship between productivity and rhythms continuously and throughout the semester. In this study, we incorporated the subjective assessments of daily productivity provided by students through evening surveys. Such survey-based methods are widely recognized in academic research as a standard approach to measure productivity, as evidenced by studies such as Tesserae [44], TILES [45], and RAAMPS [46]. It is worth noting that while subjective measurements might introduce biases, our data indicated that students maintained consistency in their responses over several weeks. Furthermore, by creating individual models for each student’s rhythms, we successfully accounted for week-to-week variations, allowing us to assess the relationship between these rhythms and the reported productivity, even considering potential biases. Overall, we were able to test our methods on this data. However, a larger and more longitudinal data set is needed to fully characterize biobehavioral rhythms from mobile data streams and model their relationship with different outcomes.

Given the observational nature of collecting sensor data unobtrusively “in the wild,” it is impossible to account for all variables that may impact the data. However, we have taken steps to qualify the potential limitations and strengthen the validity of our digital phenotyping approach within reason. We also suggest further research incorporating both subjective self-reports and sensor data to better characterize confounding contexts. With these caveats articulated, we believe our study maintains substantial value in demonstrating the promise of modeling multidimensional digital phenotypes through passively collected mobile sensor data to advance biobehavioral research.

### Conclusion

We explored the feasibility of modeling biobehavioral rhythms from longitudinal multimodal mobile data streams, focusing on college students to identify the relationship between these rhythms and productivity levels. We introduced a multidimensional correlation method to analyze connections between variations in biobehavioral rhythms and productivity. This approach enabled us to observe differences in the longitudinal behavior of high and low-productive students and highlighted that highly productive students encompass more rhythm stability throughout the semester despite variations in workload during different periods. We plan to further evaluate by testing the applicability and adaptability of our methods with diverse data sets. This research paves the way for novel cyber-human systems that align with human beings’ biobehavioral rhythms to improve health, well-being, and work performance.

## Appendix

Multimedia Appendix 1 Correlation between productivity and each sensor feature.

Multimedia Appendix 2 Correlation between productivity and each rhythm parameter.

## Abbreviations

Amp: amplitude
IP: integrated *P* value
IPR: integrated percent rhythm
MESOR: Midline Estimating Statistic of Rhythm
PHI: acrophase
PR: percent rhythm
