# Supplementary material for: Identifying Links Between Productivity and Biobehavioral Rhythms Modeled From Multimodal Sensor Streams: Exploratory Quantitative Study
Source: JMIR AI. 2024 Apr 18;3:e47194. doi: 10.2196/47194 (PMC11066747; doi:10.2196/47194)
Supplement: Multimedia Appendix 1 [file ai_v3i1e47194_app1.docx]

Let $SF_{lj}$ denote the set of sensor features that are present for rhythm parameter $l$ in time window $j$, ${SF}_{sig,lj}$ denote the subset of ${SF}_{lj}$ with $p\leq threshold t$ ($p$ represents the level of statistical significance and typically $\leq0.05$, here $t=0.05$) for rhythm parameter $l$ in time window $j$. Let ${RF}_{kj}$ denote the set of rhythm parameters that are present for sensor feature $k$ in time window $j$, ${RF}_{sig, kj}$ denote the set of $RF$ whose 𝑝 < 𝑡ℎ𝑟𝑒𝑠ℎ𝑜𝑙𝑑 𝑡 for sensor feature $k$ in time window $j$.

For each sensor feature ${sf}_{k} (k=1\ldots K)$, we first transform the sample correlation $r_{klj}$ to Fisher’s 𝑧 score by Equation 2.

$$\begin{aligned} z_{klj}=0.5\times ln\left( \frac{1+r_{klj}}{1-r_{klj}} \right)\#\left( 2 \right) \end{aligned}$$

, where $r_{klj}$ represents the significant Pearson correlation coefficient between rhythm parameter $l$ and productivity in time window $j$ for sensor feature $SF$ with the index of $k$. $RF_{sig}$ represent the set of all rhythm parameters with significant correlation.

Then, we calculate the aggregated correlations and significance between productivity and rhythm parameters ($rf_{l},l=1\ldots L$) within each time window $j, j = 1\ldots J$ (e.g., week 1) using Equation 3 and 4.

$$\begin{aligned} Z_{j}SF_{k}=\frac{\sum_{l\in RF_{sig,kj}} \left| z_{\mathrm{klj}} \right|}{n\left( l\in RF_{sig,kj} \right)}\#\left( 3 \right) \end{aligned}$$

$$\begin{aligned} S_{j}SF_{k}=\left( 1-2\times\sum_{l\in RF_{sig,kj}} \log\left( p_{\mathrm{klj}} \right) \right)\times\frac{n\left( l\in RF_{sig,kj} \right)}{n\left( l \right)}\#\left( 4 \right) \end{aligned}$$

These aggregated correlations and significance are denoted as $Z_{j}SF_{k}$ and $S_{j}SF_{k}$, which are then averaged across $J$ time windows with Equation 5 and 6.

$$\begin{aligned} ZSF_{k}=\frac{\sum_{1\leq j\leq J} Z_{j}SF_{k}}{J}\#\left( 5 \right) \end{aligned}$$

$$\begin{aligned} SSF_{k}=\frac{\sum_{1\leq j\leq J} S_{j}SF_{k}}{J}\#\left( 6 \right) \end{aligned}$$

, where 𝐽 represents the total number of time windows.

In the end, the aggregated 𝑧 score ($ZSF_{k}$) is transformed into the format of correlation coefficients by Equation 7. The final average correlations and significance are denoted as $CSF_{k}$ and $SSF_{k}$respectively.

$$\begin{aligned} CSF_{k}=\frac{e^{\left( 2\times ZSF_{k} \right)}-1}{e^{\left( 2\times ZSF_{k} \right)}+1}\#\left( 7 \right) \end{aligned}$$
