# Supplementary material for: Identifying Links Between Productivity and Biobehavioral Rhythms Modeled From Multimodal Sensor Streams: Exploratory Quantitative Study
Source: JMIR AI. 2024 Apr 18;3:e47194. doi: 10.2196/47194 (PMC11066747; doi:10.2196/47194)
Supplement: Multimedia Appendix 2 [file ai_v3i1e47194_app2.docx]

Similarly for each rhythm parameter $rf_{l}\left( l=1\ldots L \right)$ we first calculate the aggregated correlations and significance between productivity and sensor features $\left( sf_{k},k=1\ldots K \right)$ within each time window $j, j = 1\ldots J$, using Equation 8, 9 and 10.

$$\begin{aligned} z_{klj}=0.5\times ln\left( \frac{1+r_{klj}}{1-r_{klj}} \right)\#\left( 8 \right) \end{aligned}$$

$$\begin{aligned} Z_{j}RF_{l}=\frac{\sum_{k\in SF_{sig,lj}} \left| z_{\mathrm{klj}} \right|}{\left| \left| SF_{sig,lj} \right| \right|}\#\left( 9 \right) \end{aligned}$$

$$\begin{aligned} S_{j}RF_{l}=\left( 1-2\times\sum_{k\in SF_{sig,lj}} \log\left( p_{\mathrm{klj}} \right) \right)\times\frac{n\left( k\in SF_{sig,lj} \right)}{n\left( k \right)}\#\left( 10 \right) \end{aligned}$$

The aggregated correlations and significance are denoted as $Z_{j}RF_{l}$ and $S_{j}RF_{l}$ and then averaged across $J$ time windows using Equation 11 and 12. The final aggregated correlations and significance are denoted as $CRF_{l}$ and $SRF_{l}$.

$$\begin{aligned} ZRF_{l}=\frac{\sum_{1\leq j\leq J} Z_{j}RF_{l}}{J}\#\left( 11 \right) \end{aligned}$$

$$\begin{aligned} SRF_{l}=\frac{\sum_{1\leq j\leq J} S_{j}RF_{l}}{J}\#\left( 12 \right) \end{aligned}$$

$$\begin{aligned} CRF_{l}=\frac{e^{\left( 2\times ZRF_{l} \right)}-1}{e^{\left( 2\times ZRF_{l} \right)}+1}\#\left( 13 \right) \end{aligned}$$

, where $J$ represents the total number of time windows.
